# Organization and ELISA-Based Results of the First Proficiency Testing to Evaluate the Ability of European Union Laboratories to Detect Staphylococcal Enterotoxin Type B (SEB) in Buffer and Milk

**DOI:** 10.3390/toxins8090268

**Published:** 2016-09-13

**Authors:** Yacine Nia, Mélanie Rodriguez, Reinhard Zeleny, Sabine Herbin, Frédéric Auvray, Uwe Fiebig, Marc-André Avondet, Amalia Munoz, Jacques-Antoine Hennekinne

**Affiliations:** 1 Laboratory for food safety, ANSES, Université Paris-Est, Maisons-Alfort F-94700, France; yacine.nia@anses.fr (Y.N.); rodriguez.melanie@live.fr (M.R.); sabine.herbin@anses.fr (S.H.); frederic.auvray@anses.fr (F.A.); 2 Joint Research Centre, Directorate for Health, Consumers and Reference Materials, European Commission, Retieseweg 111, Geel 2440, Belgium; reinhard.zeleny@ec.europa.eu (R.Z.); Amalia.munoz-pineiro@ec.europa.eu (A.M.); 3 Centre for Biological Threats and Special Pathogens Biological Toxins, Robert Koch Institute, Berlin 13353, Germany; FiebigU@rki.de; 4 LABOR SPIEZ, Eidgenössisches Departement für Verteidigung, Bevölkerungsschutz und Sport VBS Bundesamt für Bevölkerungsschutz BABS, Spiez 3700, Switzerland; marc-andre.avondet@babs.admin.ch

**Keywords:** staphylococcal enterotoxin B, bioterrorism agent, proficiency test, ELISA methods

## Abstract

The aim of this work was to organize the first proficiency test (PT) dedicated to staphylococcal enterotoxin B (SEB) detection in milk and buffer solutions. This paper describes the organization of the PT trial according to EN ISO 17043 requirements. Characterization of the SEB stock solution was performed using SDS-PAGE and SE-specific ELISA, and amino acid analysis was used to assign its protein concentration. The solution was then used to prepare six PT materials (four milk and two buffer batches) at a ng/g toxin level, which included one blank and one SEA-containing milk as specificity control. Suitable material homogeneity and stability were assessed using screening and quantitative ELISAs. Among the methods used by the participants, ELISA-based methods demonstrated their efficiency for the detection of SEB in both simple and complex matrices. The results serve as a basis for further improving the detection capabilities in expert laboratories and can therefore be considered as a contribution to biopreparedness.

## 1. Introduction

Bacterial toxins represent the third most common causative agent of reported foodborne outbreaks within the European Union (EU), with staphylococcal enterotoxins (SEs) being considered as a major cause of foodborne illness due to bacterial toxins (i.e., 49% of cases over the period 2010–2014) [1,2,3,4,5]. The notifications of SEs in food matrices have been mandatory since 2005 (Commission Regulation (EC) No. 2073/2005 amended by EC No. 1441/2007). SEs are at the interface of classical biological and chemical agents. Among the 23 SEs serotypes, staphylococcal enterotoxin type B (SEB) could be used for terrorist attacks on the basis of its availability, ease of preparation, high toxicity, and/or lack of medical countermeasures. As ricin, botulinum neurotoxins, and saxitoxin, SEB is considered among the most relevant agents in the bioterrorism, for which the current preparedness within European countries should be further improved to control and to limit casualties in the case of an intentional release.

Coagulase positive staphylococci (CPS) are widespread bacteria commonly found in the environment and borne by some animals such as mammals and birds. SEB is a 28.336 kDa heat stable protein produced by enterotoxigenic strains of CPS, mainly *Staphylococcus aureus*. It is known as a causative agent of food-borne outbreaks, mainly as a result of food contaminated with *Staphylococcus aureus*. However, SEB is also toxic by inhalation and can be easily prepared from bacterial cultures without the need of specific equipment. Consequently, SEB is listed as a category B agent of potential bioterrorism risk by the Centers for Disease Control and Prevention (CDC) [6]. Additionally, SEB has a history of military use as a potential warfare agent because it can easily be aerosolized and can cause shock and death when inhaled at very high dosages. However, it is classified as an incapacitating agent [7] because in most cases aerosol exposure does not result in death but in a temporary, though profoundly incapacitating illness. The use of this agent can rapidly overwhelm the medical infrastructure if it causes large-scale incapacitation [8]. Therefore, SEB is a prohibited substance under the Biological Weapons Convention (BWC).

Since 2003, the European Union Reference Laboratory for coagulase positive staphylococci (EURL for CPS) annually organized proficiency test (PT) dedicated to SEs detection according to the EC regulation [9] but none of them focussed on SEB due to the restrictions for handling and shipment associated to its classification.

While different methods (generally based on ELISA) for SEB detection and analysis have been established, there is actually no universal SEB standard available. Generally, PTs and certified reference materials for the SEB are lacking, as well as standardized validated methods for biosecurity applications. In this context, the aim of EQuATox project was the Establishment of Quality Assurances for the Detection of Biological Toxins of Potential Bioterrorism Risk [10]. Expert laboratories from food safety and security sectors took part this project. To evaluate the capability of the EQuATox network, laboratories to properly detect SEB in food samples, PT using milk and buffer samples was organized. Participants were free to use one or more methods, in-house and/or commercially available, routine and/or recently developed in their laboratories.

This paper describes the characterization of the SEB pure material used as a spiking solution and the subsequent preparation of homogeneous and stable matrix materials as well as the overall organization of this first PT to evaluate the SEB detection capability of the participating laboratories. The paper focuses on the results obtained by the most widely used tools to detect staphylococcal enterotoxins: immunochemical assays and methods.

## 2. Results

### 2.1. Characterization of Toxins

**Figure 1:**
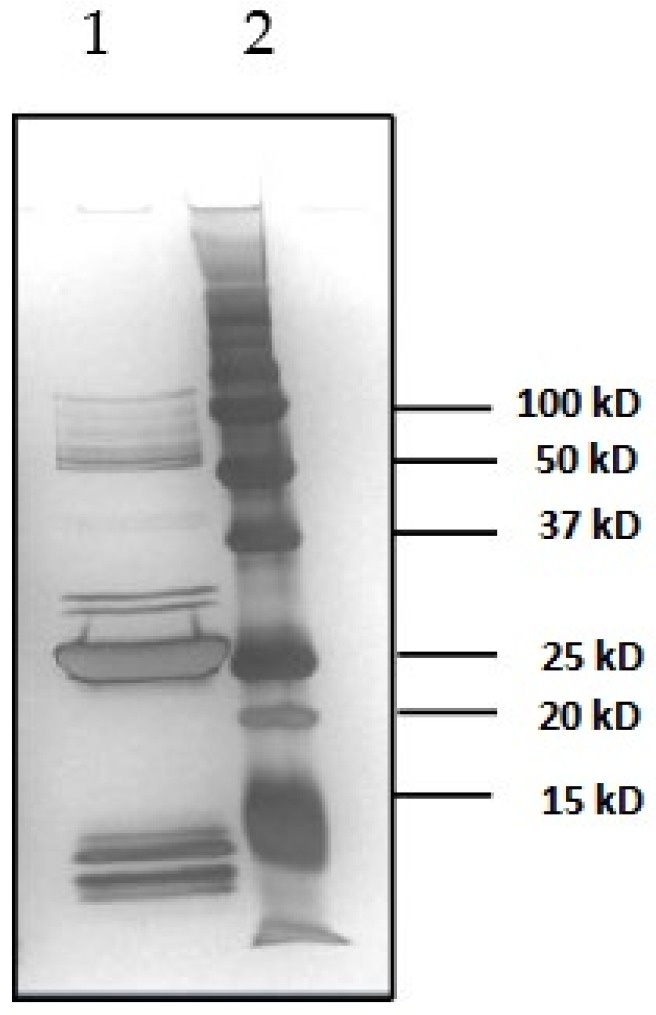
SDS-PAGE and silver staining of the SEB solution used for spiking.1: SEB solution (Toxin Technology, Sarasota, FL, USA, Ref BT202 batch number: 91709B); 2: Precision Plus Protein all blue prestained proteins standards (Biorad, Marnes la coquette, France).

Due to the lack of an available reference material for SEB, there was a crucial need to characterize and verify the purity of the commercially obtained toxin before organizing the PT trial. In this paper, we demonstrated that the SEA and SEB toxins purchased from Toxin Technology (Sarasato, FL, USA) were sufficiently pure to be used for sample preparation. This was accomplished by performing SDS-PAGE with silver staining and SEA-SEE specific ELISAs (Figure 1). Results obtained highlighted that the SEB solution used was not as pure as expected considering the quality control certificate provided by the manufacturer but toxins SEA, SEC, and SED have not been quantified by quantitative ELISA method used. However, the results confirmed that the purity was sufficiently high to use LC-ID-MS/MS amino acid analysis (AAA) for establishing a protein quantity value from the SEB solution, and that this solution was fit-for-purpose to be used as spike for the preparation of suitable matrix PT materials.

#### 2.1.1. Determination of SEB Protein Concentration by in-House LC-ID-MS/MS AAA

The classical approach of protein quantification by UV-spectrometry and published extinction coefficients is unspecific (absorbance value only) and thus often questionable. Therefore, protein quantification was performed using isotopic dilution mass spectrometry (IDMS) as described elsewhere [11]. SEB was completely hydrolyzed into its principal components, the amino acids. Subsequently, the six most acid stable amino acids (alanine, proline, valine, leucine, isoleucine, and phenylalanine) were quantified based on IDMS. Knowledge of the purity and amino acid sequence composition of the protein of interest allows calculation of the results. The calculations are based on the quantity of individual amino acids present in the solution. The protein to be analyzed with this method needs to be sufficiently pure, as otherwise the obtained AAA result cannot be related to the theoretical sequence of that protein and, thus, are meaningless since the method cannot differentiate whether a quantified amino acid stems from different proteins in the original sample. Six independent replicate analyses were performed under repeatability conditions. The obtained result was 0.125 ± 0.007 mg SEB/mL solution. As the analyzed solution was a carefully prepared 10-fold dilution of the provided stock solution, the SEB content in the stock was thus calculated to be 1.25 ± 0.07 mg/mL. The resulting variability is expressed as expanded uncertainty (coverage factor 2), incorporating contributions from repeatability, intermediate precision, calibration, volumetric dilutions, and among-amino acid variability.

#### 2.1.2. Determination of SEB Protein Concentration by HPLC-FLD of OPA-Derivatized Amino Acids

Three independent samples were prepared and analyzed in duplicate under repeatable conditions. The result obtained was 0.136 ± 0.004 mg/mL. As the analyzed solution was a carefully prepared 8-fold dilution of the stock solution, the SEB content in the stock was thus 1.09 ± 0.03 mg/mL. The results variability reflects measurement repeatability, but not measurement uncertainty as established for the other method (see uncertainty contributors above, lines 92–94). As robust estimate of the protein concentration in the SEB stock solution, the mean of results obtained by AAA and HPLC-FLD methods was taken, amounting to 1.17 mg/mL. This value was used for spiking the PT trial samples.

#### 2.1.3. Determination of SEA Protein Concentration by PSAQ Technology

As the SEA solution used for spiking one of the matrix materials in this PT was previously quantified by the Protein Standard Absolute Quantification (PSAQ) method [12], we did not perform AAA analysis and used the PSAQ value equal to 0.506 mg/mL to prepare milk sample containing SEA.

### 2.2. PT Materials Preparation, Homogeneity, and Stability Assessment

Materials were prepared as outlined in the materials and methods section, and were checked for suitable homogeneity before dispatch to the laboratories (PT round). These results were compared to those obtained from analysis of randomly selected samples after receiving all the results from the participants in order to evaluate stability (see below).

Characterization of materials therefore included both homogeneity and stability studies.

**Figure 2:**
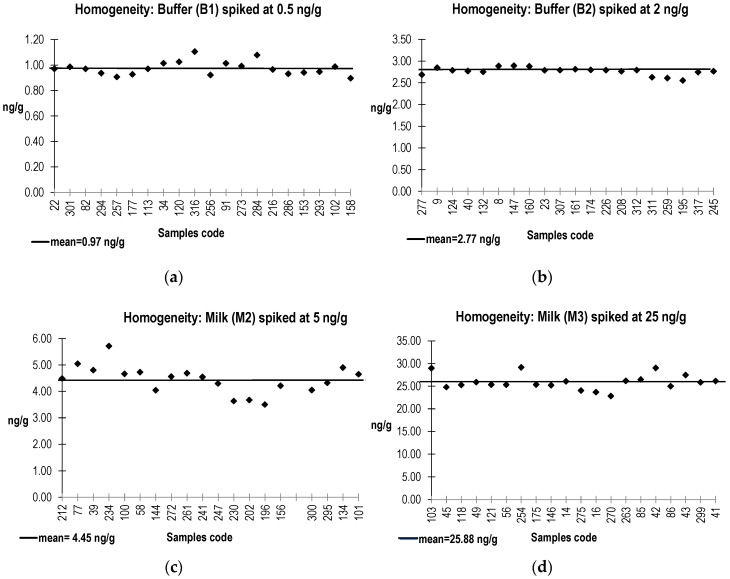
Homogeneity data obtained for the buffer (B1 and B2) and milk (M2 and M3) samples spiked by SEB. (**a**): in buffer (B1), (**b**): in buffer (B2), (**c**): in milk (M2), (**d**): in milk (M3).

Homogeneity data for buffer (B1 and B2) and milk (M2 and M3) samples spiked with SEB are presented in Figure 2.

For the PT material M1 (blank milk), the applied European Screening Method (ESM) using the VIDAS SET2 detection confirmed the absence of the staphylococcal enterotoxins types SEA, SEB, SEC, SED, and SEE in each of the 20 individual samples analyzed.

SEB was analyzed in PT buffer (B1, B2) and milk (M2, M3 and M4) materials by the in-house quantitative ELISA (limit of detection: 0.06 ng/mL). For materials B1, B2, M2, and M3 spiked by SEB, the Relative Standard Deviation (RSD) from 20 individual samples ranged from 3% to 12%. As these values were lower than 15% (value commonly admitted to define homogeneity in the case of ELISA based method), milk (M2, M3) and buffer (B1, B2) samples were considered as homogenous. For PT material M4 spiked by SEA, SEB was not detected in the 20 individual samples.

**Figure 3:**
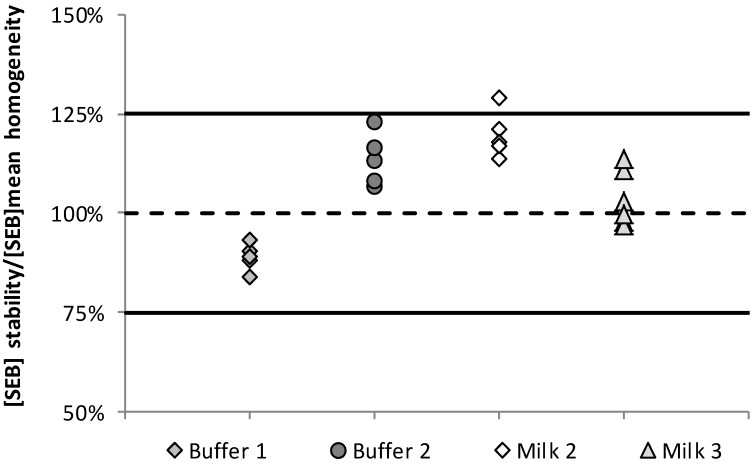
Stability data obtained for the milk and buffer samples spiked by SEB. Data were presented as recovery calculated from the measured SEB concentrations in stability samples and the assigned value corresponding to the homogeneity value.

The homogeneity test was performed on 20 samples one week before samples dispatch. In order to assess to stability of SEB in each batch (M1 to M4, B1 and B2), six randomly selected samples were analyzed after receiving participant results (six weeks after dispatching). Then, these six replicates were compared to the mean of the 20 values obtained in the homogeneity study (Equation (1)). Results are presented in Figure 3.

(1) $$\left[\mathrm{Buffer}\;1\;\mathrm{recovery}\right]\%=\frac{\mathrm{SEB}\;\mathrm{concentration}\;\mathrm{measured}\;\mathrm{in}\;\mathrm{Buffer}\;1\;\left(\mathrm{stability}\right)}{\mathrm{the}\;\mathrm{assigned}\;\mathrm{value}\;\mathrm{for}\;\mathrm{Buffer}\;1\;\left(\mathrm{homogeneity}\right)}\times 100$$

All the data but one replicate (M2 at +29%) were included between lower and upper limits defined for stability measurement (±25%) (Figure 2). As the aim of this stability study was to demonstrate that SEB must be detectable over the PT trial period, we considered that this outlier value (+29%) did not affect the appreciation of samples stability. Therefore:

(a) SEB in the six replicates dedicated to the stability test were well detected and quantified in buffer (B1 and B2) and milk (M1 and M2) samples, allowing the consideration that the samples were sufficiently stable when stored at 5 ± 3 °C over the PT period.
(b) For material M4 spiked with SEA, SEB was not detected in the six replicates confirming the absence of cross reactions between the SEA and SEB using the in-house quantitative ELISA.
(c) As in the homogeneity tests, six milk blank samples (M1) were analyzed by the ESM using the VIDAS SET2 detection assay. Data obtained showed that the five SE types (SEA, SEB, SEC, SED, and SEE) were not detected, confirming the absence of SEB external contamination in this blank material (M1).

### 2.3. PT Trial Organization

The PT trial was organized in five major steps:

(a) laboratories from the network were invited to participate the PT trial and received information on the dispatch of samples and the period dedicated for analyzing samples and reporting results (six weeks).
  17 participants confirmed their participation using the ELISA method, but 1 laboratory withdrew after receiving the samples. Therefore, 16 laboratories took parts in this PT trial using the ELISA methods.
(b) samples were prepared (Section 5.3) and homogeneity test was performed on 20 replicates for each material.
(c) (once the homogeneity was confirmed, samples were dispatched on the indicated period, but only to participants registered for the test.
(d) after receiving the participant results, a stability test was performed for each material on the six replicates dedicated to this test (Section 5).
(e) received data were assessed, a report was produced and participants received their individual results.

### 2.4. Results Obtained by ELISA-Based Methods

15 laboratories provided results with one or more qualitative ELISA based methods (Table 1). A color-code was used for interpretation:

(a) A green box: Staphylococcal enterotoxins were detected, the serotype SEB was identified in samples B1 and B2, M2 and M3. For M1 sample (blank), SEs were not detected especially SEB. For M4 sample (SEA spiked), SEB was not specifically detected.
(b) A light green box: Staphylococcal enterotoxin type SEB was detected in B1, B2, M2, and M3 but other serotypes were identified in these samples. For M1 sample (blank) and M4 samples (SEA spiked), SEs were not detected without specification of the toxin type.
(c) A red box: false positive or false negative results were obtained.
(d) A white box: samples were not analyzed.

As this study aimed to check the capability of the network to detect SEB, participants were free to use their own method, based on internal reactive or commercially available detection assays.

In this PT Trial, several qualitative and quantitative in-house ELISA methods were used. Also, eight commercially available detection assays were tested, including Vidas SET2 (Biomerieux, Marcy l’Étoile, France), Ridascreen SET ABCDE (Rbiopharm, Darmstadt, Germany), Ridascreen SET Total (Rbiopharm), Tecra SET VIA (3M, Roseville, New South Wales, Australia), Miprotect SEB (Miprolab, Göttingen, Germany), SET- RPLA (OXOID, Hampshire, UK), TetraCore ELISA kit (TetraCore, Rockville, MD, USA), and Portable Toxin Detector pTD (Bruker Daltonik, Leipzig, Germany).

In general, no false positive results were obtained by the network. However, one false negative result was obtained for M2 (5 ng/g), and five false negative results for B1 (0.5 ng/g). In addition, among 15 laboratories that used qualitative ELISA (including in-house and commercial assays), 10 laboratories obtained 100% correct results (detection of the serotype SEB by at least one detection assay), and five laboratories identified correctly the presence of staphylococcal enterotoxins but did not identify the serotype (i.e., SEB) by at least one detection assay.

Nine laboratories also used quantitative ELISA methods (Table 2) to evaluate the SEB content of the provided samples.

These results highlighted that the methods used by laboratory coded as 3, 9, 12, and 17 have reliably quantified SEB in a buffer solution as well as in a complex food matrix. Three out of the 9 laboratories detected and quantified SEB in samples (and SEA in M4), as shown by their *Z* score ≤ 2.0. In contrast, the method used by laboratory number 16 provided inconsistent quantitative results compared to the expected ones.

The quantitative assessment showed that the quantitative ELISA methods are the most sensitive. These are developed generally by the participants using in-house or commercially available antibodies.

## 3. Discussion

Generally, the most commonly used method for SEs detection in food matrices is based on the use of anti-enterotoxin polyclonal or monoclonal antibodies. Commercially available assays have been developed according to two principles:

(a) enzyme immunoassay (EIA) comprising enzyme-linked immunosorbent assay (ELISA) and enzyme-linked fluorescent assay (ELFA).
(b) reversed passive latex agglutination (RPLA).

The difficulties related to immunological methods for contaminant detection in food matrices were widely recognized, mainly due to the lack of specificity and sensitivity of the available assay. Many drawbacks impact the development and use of these techniques for SEs detection in food matrices. First, highly purified toxins are necessary to produce specific antibodies to develop an EIA; but they are difficult and expensive to obtain. Also, some commercial kits show a low specificity where the false positives may occur depending on matrix components as it is well known that some proteins (e.g., protein A), can interfere with binding to the Fc fragment (and, to a lesser extent, Fab fragments) in immunoglobulin G from several animal species (e.g., mouse or rabbit, but not rat or goat). Other interferences are associated with endogenous enzymes, such as alkaline phosphatase or lactoperoxidase. This paper focuses on the use of such methods to detect SE content in the provided samples.

Due to drawbacks with currently available detection methods, other strategies based on physico-chemical techniques have been developed very recently. Among these, mass spectrometry (MS) has emerged as a very promising and suitable technique for analyzing protein and peptide mixtures [16]. The development of two soft ionization methods, such as electrospray ionization (ESI) and matrix-assisted laser desorption/ionization (MALDI), and the use of appropriate mass analyzers such as time-of flight (TOF), have revolutionized the analysis of biomolecules.

In the case of food analysis, the situation is complex because the matrix can contain many proteins, lipids and many other molecular species that interfere with the detection of the targeted toxin and may distort quantification. Sample preparation remains the critical step of the analysis. Few participating laboratories developed and used mass-spectrometry based method. Even if this work focused on ELISA method, few laboratories participated to the PT Trial using liquid chromatography coupled mass spectrometry (LC-MS). SEA and SEB in buffer and milk samples were extracted, purified, and were digested using Trypsin. Then, the purified extract was analyzed by LC-MS. In fact, SEB specific peptides were separated in the chromatography column and detected by the MS tool. Among these methods, one was sufficiently specific to discriminate SEB from other SE types in milk sample but not sensitive enough to prove SEB presence in buffer solution [17].

The PT samples were prepared according to ISO 17043 and following an internal procedure used for several years at the EURL for CPS when organizing PT trials among NRLs [18]. The only difference in the EQuATox PT study was that materials were stored refrigerated and not frozen. The stability study results described above underline the validity of this approach. Moreover, the acquired homogeneity and stability results on SEB in these matrix materials point at the possibility for preparing suitable non-frozen matrix materials for future PT studies, both in the food safety field (“all” SEs), as well as for biosecurity purposes (SEB detection capability of laboratories). Finally, the obtained stability data for SEB provides valuable information for assessing the feasibility for a certified reference material (CRM) for this toxin in a suitable matrix. Currently, only one CRM, a freeze dried cheese, containing SEA at two levels was produced recently and is available to the scientific community [19].

Considering the results obtained, the use of commercially available assays allowed reliable detection of SEB in the tested samples in most cases. Some of them were able to differentiate SEB from other toxin types whereas others were not. These results highlighted that cross reactions could occur with the use of two commercially available detection kits which has been already demonstrated [20]. Moreover, analysis of quantitative results highlighted that various in-house methods have the potential for a reliable quantification of SEB in a buffer solution as well as in a complex food matrix, providing increased flexibility to the laboratories for enterotoxin quantification.

Some of the commercially available detection methods as well as some of in-house ELISAs demonstrated their capability to specifically detect and/or quantify SEB in the various types of samples.

## 4. Conclusions

The results obtained in the SEB PT trial, dealing with the evaluation of the capability of the EQuATox network laboratories to properly detect SEB in a buffer as well as in a complex food matrix will contribute to a better understanding and further improvement of the available methods for reliable detection of this potential bioweapon.

This was accomplished by the preparation of homogeneous materials that showed suitable stability for the time frame of the study when stored under refrigerated condition. The majority of data sets obtained were acquired by using immunochemical methods, especially ELISAs. Several of those ELISAs are commercially available, and some of them allow distinction among SE types SEA-SEE, whereas others do not allow this distinction and can therefore only be used as screening tools. Concerning accurate quantification of SEB in buffer and milk samples, some in-house ELISAs showed suitable performance in a number of laboratories. If properly validated, some of these specific methods could be made available to designated laboratories in charge of the detection of SEB in various types of samples. Finally, the described results are valuable to support the development of suitable CRMs for SE detection in complex matrices.

## 5. Materials and Methods

### 5.1. Choice of Samples

Two types of matrices were selected for this PT:

(a) Phosphate buffered saline (NaCl/Na_2_HPO_4_: 145 mM/10 mM, Sigma-Aldrich (St. Quentin Fallavier, France)) at pH 7.3 ± 0.2 was considered as an easy to use matrix.
(b) As the EU regulation 2073/2005 modified by the European regulation 1441/2007 [21] enforces testing of SEs content in milk and milk based products, it was decided to use liquid UHT fat-free milk as a complex matrix (purchased from a retail store).

Before starting the samples preparation, the milk matrix was tested for staphylococcal enterotoxin types SEA to SEE using quantitative ELISAs specific for each of the five toxin types. The results confirmed the non-detection of SEA–SEE in the milk (<LOD of method). Moreover, a SEA-spiked liquid milk sample has also been prepared to evaluate the specificity of the methods used by participants.

### 5.2. Characterization of Toxins

Highly purified freeze-dried SEB (Toxin Technology, Sarasota, FL, Ref BT202 batch number: 91709B) was rehydrated with osmosis water according to the manufacturer’s instructions to obtain a stock solution with a nominal concentration of 1 mg/mL. Purity of the SEB was assessed after migration on a 10% sodium dodecyl sulfate polyacrylamide gel electrophoresis (SDS-PAGE) and stained with Silver nitrate.

#### 5.2.1. Determination of Protein Concentration of SEB by Amino Acid Analysis (AAA)

In order to characterize and to assign a protein concentration for the SEB stock solution, two samples were independently characterized by amino acid analysis (AAA) considering a molecular weight (MW) equal to 28,336 Da [22] in two laboratories:

(a) SGS M-Scan (SGS M-Scan; Freiburg, Germany) [23]. Briefly, Norvaline and Sarcosine were added to SEB solution as internal standards for primary and secondary amino acids and hydrolyzed in 6 M HCl at 110 °C for 24 h. Then, the obtained solution was analyzed by HPLC with fluorescence detection following pre-column derivatization with OPA (ortho-phthalaldehyde) with fluorescence detection at 340/450 nm for excitation and emission, respectively. For calculation of protein concentrations, only robust amino acids were taken into account as follows;
(b) JRC-IRMM, Geel, Belgium (from EQuATox network). AAA was carried out as described in detail in the laboratory working instruction of JRC-IRMM and is based on the publication by Muñoz et al. [11]. Briefly, the protein sample is hydrolyzed using 6 M HCl and 0.1% of phenol using a 1 h microwave digestion protocol. Liberated amino acids were separated on a reverse-phase HPLC column and quantified by tandem mass spectrometry using pure amino acids for calibration and isotopically labelled amino acids as internal standards. Ala, Pro, Val, Leu, Iso, and Phe are the amino acids which are most stable during hydrolysis and resistant to oxidation under the conditions employed; these were thus quantified and the results converted into protein concentration using the published SEB protein sequence [22]. Some deviations from the validated method were made, for instance: volumetric instead of gravimetric sample preparation due to safety reasons; employment of lower volumes of toxin, buffer, and internal standard solutions to allow six independent analyses; and the omission of a so-called sample blank (sample with all solvents, reagents and internal standards, but without hydrolysis, again for safety reasons).

However, a so-called reagent blank (solvent, reagents, but no calibrant amino acids and internal standard isotopically-labelled amino acids; sample undergoing digestion) was included as QC sample to check for the absence of free amino acids and labelled amino acids in that sample. Moreover, a second QC (solvent and labelled amino acids, but no calibrant amino acids; sample undergoing digestion) was included to check for integrity of the internal standard solution (absence of free amino acids in this solution).

#### 5.2.2. Determination of Protein Concentration of SEA by PSAQ Technology

Highly purified staphylococcal SEA (Technology, Sarasota, FL, USA, Ref AT101, batch number 120794A) used to prepare the M4 sample was previously quantified by nanoLC-ESI-MS/MS using protein standard absolute quantification (PSAQ) technology [24].

### 5.3. Preparation of Samples

**Figure 4:**
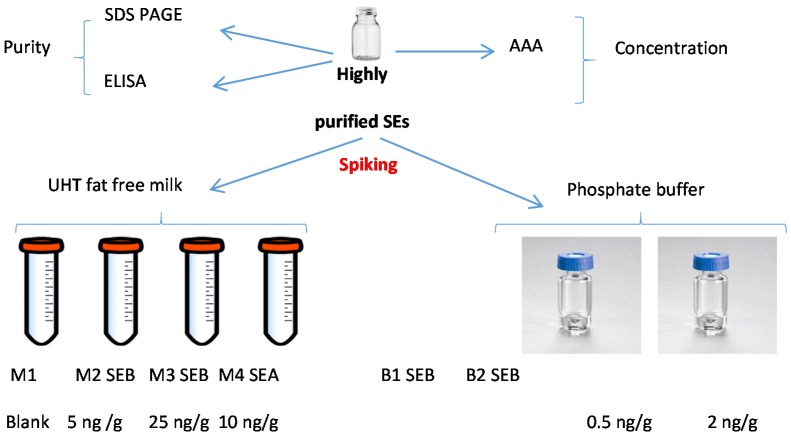
Preparation of the SEB samples.

EURL protocols were used to organize PT trials for the detection of SEs in food matrices in order to evaluate the capability of EU Member State laboratories to satisfactorily apply the EU regulation. These PT trials are generally organized using frozen food matrices as it is well known that SEs are stable. In order to evaluate the stability of SEB in liquid matrices stored at 5 ± 3 °C (envisaged storage conditions for this PT), preliminary experiments were performed. As these results obtained were satisfactory in terms of homogeneity and stability, we decided to prepare six batches for the PT trial:

(a) blank milk sample (M1),
(b) two milk samples spiked with SEB at 5 ng/g (M2) and 25 ng/g (M3),
(c) one milk sample spiked with SEA at 10 ng/g (M4),
(d) two buffer samples spiked with SEB at 0.5 ng/g (B1) and 2 ng/g (B2) (Figure 4).

All materials have been prepared according to the requirements of the EN ISO 17043 Standard [25]. For each material, a sufficient number of samples was prepared to perform:

(a) homogeneity (20 samples for each material) and stability studies (6 samples for each material),
(b) dispatch samples dedicated to participants,
(c) additional samples in case of need (10 samples for each material).

Each level was separately prepared to avoid cross contamination.

For each material, 1.4 L of milk were spiked at the targeted SE type/concentration, and aliquoted in individual containers under sterile conditions, with 25.0 g ± 0.1 g per container. During the spiking step, it was assumed that 1 mL of milk corresponded to 1 g. In total, 53 containers of 25.0 mL including homogeneity samples (20 samples), stability samples (6 samples), samples for participants, and additional samples were prepared for each milk material (M1, M2, M3, and M4).

In addition, as for milk samples, a Phosphate buffer/BSA/azide solution was prepared, spiked with SEB solution and dispatched in individual containers with 1 mL per container. In total, 53 containers of 1 mL of each buffer material (B1 and B2) were prepared.

Finally, all containers (participants, homogeneity, stability, and additional samples) were stored at a temperature of 5 ± 3 °C during the test period. Random codification was used for samples to be dispatched to participants.

### 5.4. Homogeneity Study and Determination of Assigned Values

According to EN ISO 13528 [26], 20 containers were randomly taken from each of the six batches prepared (M1, M2, M3, M4, B1, and B2). Each sample was quantitatively analyzed once with the quantitative ELISA developed in the EURL for CPS except for the blank (M1), which was analyzed by the so-called European Screening Method (ESM) [18].

The EURL for CPS has been accredited for SEs detection in food matrices according to the EN ISO CEI 17025 Standard (accreditation scope No. 1-2246) [27]. For sample preparation (homogeneity and stability tests), an extraction step was performed according to the ESM. Briefly, all milk samples were first submitted to a protein extraction step followed by a dialysis concentration step overnight against 30% PEG. Then, the obtained extract was analyzed by:

(a) the quantitative ELISA (M2, M3, and M4), which allowed to estimate the concentration of SEB in M2 and M3 samples, and to check cross reaction in the case of M4 sample (spiked by SEA).
(b) the Vidas SET2 detection kit (BioMerieux, Marcy l’Étoile, France), which is able to detect SEA to SEE in food matrices. Only blank sample M1 was analyzed, in order to check the absence of contamination of the milk by the 5 SE serotypes (i.e., SEA, SEB, SEC, SED, and SEE) [28,29].

The detection was directly performed on the buffer samples without performing the dialysis concentration step. When the ESM results were positive, the extracts obtained were submitted to the in-house quantitative ELISA confirmatory method for SEA to SEE [15].

A single sandwich type was used for SEB whereas double sandwich type ELISA was used for SEA. For the detection step, specific commercially available antibodies (Toxin Technology, Sarasota, FL, USA) were used as coating (ref SLAI101 and SLBI202 for SEA and SEB, respectively) and probing antibodies (ref LAI101 and LBC202 for SEA and SEB, respectively). The presence of toxins was revealed by immunoglobulins coupled to horseradish peroxidase (ref LBC202 for SEB, and goat anti-rabbit peroxidase labelled IgG antibodies, KPL) and determined by a colorimetric measurement at 405/630 nm after addition of ABTS (KPL).

The homogeneity acceptance criterion was defined as follows: 100% of the obtained results above the LOQ of the quantification method used and a relative standard deviation (RSD) less than or equal to 15%. For blank and SEA-spiked milk samples (M1 and M4, respectively), 100% of the results obtained should be below the LOQ of the quantification method used.

### 5.5. Stability Study of Batches

According to the EN ISO 13528, at least, six replicates per batch were analyzed after receiving all the participant results. Then, these data were compared to the homogeneity data (performed before sending samples to participants) in order to check the stability of SEB for the six batches over the analysis period (6 weeks).

The blank milk (M1) and SEA-spiked milk (M4) samples were considered as stable if the values obtained during the study period were below the positive threshold of the kit used.

For SEB contaminated samples (B1, B2, M2, and M3) and for each stability point, the calculated RSD on the six replicates should be below 15%. Values obtained during the stability studies, for each material, should be equal to the assigned value (corresponding to the homogeneity value) ± 25%. This 25% value is used by the EURL for CPS in the frame of its PT trial organization and corresponds to repeatability and reproducibility conditions during the stability testing period.

### 5.6. Organization of Proficiency Testing

Sixteen laboratories representing 13 EU-countries took part in this SEB proficiency test. The participant laboratories were invited to use the qualitative, and/or quantitative method(s) currently used in-house. Most of the participants used ELISA-based methods as these ones are commonly used to detect and/or quantify proteins from various types of samples.

Due to its classification as dangerous material (IATA 6.1 class), the samples were shipped to each of the laboratories only upon receiving all the authorizations for the SEB export to each laboratory. Six encoded samples stored at 5 ± 3 °C were shipped under security procedures by World Courier under refrigeration at positive temperature. Instructions for participants, a report sheet for results, and an acknowledgment of receipt were included in the parcels. All the samples had been received by participants within 48 h after dispatching.

## Figures and Tables

**Table 1:** Evaluation of the ELISA based qualitative results obtained by participating laboratories.

Lab Code	Method Used		M1	M2	M3	M4	B1	B2
1	ELISA electrochemical immunosensor—Portable Toxin Detector pTD (Bruker)							
3	Ridascreen SET ABCDE (Rbiopharm)							
	In house ELISA							
4	Vidas SET2 (Biomerieux)							
5	Oxoid SET RPLA (Termo Scientific)							
6	Vidas SET2 (Biomerieux)							
	Oxoid SET RPLA (Termo Scientific)							
	TetraCore ELISA kit (TetraCore)							
7	miPROTECT SEB (Miprolab)							
8	In house ELISA							
	Western blot							
9	In house ELISA [13,14]	extraction without dialysis concentration						
		extraction with dialysis concentration						
10	miPROTECT SEB (Miprolab)							
11	Vidas SET2 (Biomerieux)							
	Oxoid SET RPLA (Termo Scientific)							
12	Vidas SET2 (Biomerieux)							
	Tecra SET VIA (3M)							
	Ridascreen SET Total (Rbiopharm)							
	In house ELISA [15]							
13	Vidas SET2 (Biomerieux)							
	Oxoid SET RPLA (Termo Scientific)							
14	Ridascreen SET ABCDE (Rbiopharm)							
	TetraCore ELISA kit (TetraCore)							
	In house ELISA							
16	Lateral Flow Assay							
17	In house EIA							
	In house Immunochromatographic Test SEB							

A green box: SEB identified in samples B1 and B2, M2 and M3. For M1, SEB specifically not detected. For M4, SEB not specifically detected, A light green box: SEB detected in B1, B2, M2, and M3 but other serotypes were identified in these samples. For M1 and M4, SEs were not detected without specification of the toxin type, A red box: false positive or false negative results were obtained, A white box: samples were not analyzed.

**Table 2:** Evaluation of quantitative results obtained by ELISA-based methods.

Lab Code	Method Used		M2	M3	B1	B2
1	ELISA electrochemical immunosensor—Portable Toxin Detector pTD (Bruker)					
3	In house ELISA					
7	miPROTECT SEB (Miprolab)					
8	In house ELISA					
9	In house ELISA [13,14]	extraction without dialysis concentration				
		extraction with dialysis concentration				
12	In-house-ELISA [15]					
14	In-house-ELISA					
16	Lateral Flow Assay					
17	In house EIA					
17	In house Immunochromatographic Test SEB					

Interpretation: |Z|≤2.0: satisfactory results; 2.0<|Z|<3.0: questionable results; |Z|>3.0: unsatisfactory results.
